# Solving dynamical systems in neuromorphic hardware: simulation studies using balanced spiking networks

**DOI:** 10.1186/1471-2202-14-S1-P382

**Published:** 2013-07-08

**Authors:** Anna S Bulanova, Olivier Temam, Rodolphe Heliot

**Affiliations:** 1 ByMoore, INRIA Saclay, Palaiseau, 91120, France; 2 CEA - LETI, Minatec Campus, Grenoble, 38054, France

## 

We aim at efficiently implementing and solving linear dynamical systems using neuromorphic hardware. For this task we used Deneve's balanced spiking network framework [1]. In this framework, recurrent spiking network of Leaky Integrate-and-Fire (LIF) neurons can track solution of a linear dynamical system by minimizing prediction error; weighted leaky integration is used to decode spike trains into a continuous signal. These networks have the following properties similar to properties of real biological networks: high trial-to-trial variability, asynchronous firing, tight balance between excitation and inhibition. Additionally, such networks could be implemented in silicon using analog neurons [2].

Analog neurons are power and cost-efficient, since they directly take advantage of physics laws. Noise induced during each analog operation is not cascaded but suppressed when a logical spike is emitted at the neuron's output. This hybrid scheme results in low area and power constraints for the neuron design while preserving computation efficiency. Creating neuromorphic microchips solving linear dynamical systems can greatly expand application scope of neuromorphic hardware, currently largely used for pattern recognition tasks. With some limitations of hardware implementations in mind, we set the following constraints for the network: 1) limited firing rate to keep energy cost low, 2) connection weights need to be set in advance (no learning), 3) the network should track the solution with a required precision, 4) network contains 1000-10000 of neurons.

Our goal was to find out how to set the network parameters to achieve required solution accuracy. We considered several real life examples of dynamical systems. To learn how different network parameters affect accuracy of the solution, we ran Brian/Python simulations with different weight matrices, network noises, simulation time steps and leak constants. In this kind of networks, with each spike, the estimate of the solution is corrected roughly by the corresponding neuron's output weight vector. Testing showed that the network produces much more accurate results if the output weight vectors of all neurons have approximately equal euclidean norm. These two facts would cause problems with dynamical systems in which system variables exhibit different dynamics. The solution is to scale the variables of the dynamical systems using a linear transform chosen according to the following requirements: 1) the result of each spike should be large enough relative to the solution size so that the network can keep up with all variables' rate of change and decoder leaks under the condition of limited firing rate, 2) it should be small enough to avoid large relative error, 3) firing thresholds should be small enough compared to the input so that neuron leaks don't create a large error.

We found that the network performs well with a reasonable amount of network noise, and, as expected, performance increases with the number of neurons. In our tests, normalized root-mean square error was under 0.01. These results have real-life applications, such as systems observation.
